# Developmental delay in congenital hypothyroidism

**DOI:** 10.11604/pamj.2021.38.165.27321

**Published:** 2021-02-12

**Authors:** Chanan Goyal, Waqar Naqvi

**Affiliations:** 1 Datta Meghe Institute of Medical Sciences, Wardha, Maharashtra, India,; 2 Community Health Physiotherapy, Ravi Nair Physiotherapy College, Datta Meghe Institute of Medical Sciences, Wardha, Maharashtra, India

**Keywords:** Congenital hypothyroidism, cretinism, developmental delay

## Image in medicine

We report the case of a 5-month-old male, born out of a nonconsanguineous marriage, who presented with an inability to hold neck. As per the parents, he had diminished spontaneous movements since birth and described him as lethargic. He was clinically diagnosed with cretinism by a pediatric neurologist. He was referred by to the department of physiotherapy for evaluation and advice for the associated motor delay. On observation, the infant displayed peculiar clinical features of sparse hair, periorbital edema and macroglossia (A). Irritability, hoarse cry, abdominal distension and frog leg position was observed in supine lying position (B). Examination revealed generalized hypotonia, wide fontanelles, feeding difficulties and global developmental delay. He was neither able to roll over from supine to prone nor was he able to hold neck adequately. He had head lag during pull to sit test. Due to financial constraints, they were not willing to attend physiotherapy sessions on a regular basis. Thus, parents were educated about the home exercise program that included active assisted rolling, neck extensor muscles facilitation against gravity while lying prone on forearm, active assisted reaching out for objects while lying, neck flexor muscles facilitation in gravity minimized plane and active neck rotations to look at objects of interest while side lying on forearm support. Congenital hypothyroidism is still a common cause of developmental delay in newborns of the developing countries. Awareness, universal newborn screening, prompt recognition and medical treatment along with physiotherapy can minimize adverse effect on the physical and neurocognitive development.

**Figure 1:**
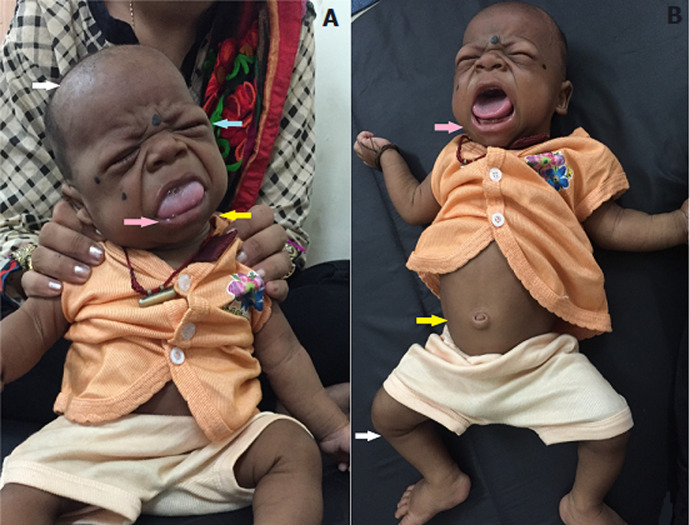
clinical features of congenital hypothyroidism or cretinism: A) white arrow shows sparse hair, blue arrow shows periorbital edema, pink arrow shows macroglossia and yellow arrow points towards lack of head control; B) pink arrow points towards irritability and hoarse cry, yellow arrow shows flabby abdomen and white arrow shows frog leg position due to hypotonia

